# Level of job satisfaction and associated factors among health care professionals working at University of Gondar Referral Hospital, Northwest Ethiopia: a cross-sectional study

**DOI:** 10.1186/s13104-018-3918-0

**Published:** 2018-11-20

**Authors:** Genet Gedif, Yetnayet Sisay, Animut Alebel, Yihalem Abebe Belay

**Affiliations:** 1 Department of Public Health, College of Health Sciences, Debre Markos University, P.O.BOX:269, Debre Markos, Ethiopia; 2 Department of Nursing, College of Health Sciences, Debre Markos University, Debre Markos, Ethiopia; 3 Department of Health Education and Behavioral Science, Institute of Public Health, University of Gondar, Gondar, Ethiopia

**Keywords:** Associated factors, University of Gondar, Health care professionals, Job satisfaction, Referral hospital

## Abstract

**Objectives:**

The main aim of this study was to assess the level of job satisfaction and associated factors among healthcare professionals working at University of Gondar Referral Hospital, Northwest Ethiopia. An institution based cross-sectional study was conducted among 416 healthcare professionals from March 27, 2017 to April 25, 2017. Simple random sampling technique was employed and data were collected with a pre-tested interviewer administered questionnaire. Data were entered into Epi-Info version 7, and analyzed using SPSS 20 softwares. Binary logistic regression analysis was employed.

**Results:**

A total of 383 participants were involved in the study. The overall level of job satisfaction among health care professionals was 54% [95% CI (49.3–58.8)]. Marital status [AOR = 1.79 (1.140, 2.797)], salary [AOR = 2.75 (1.269, 5.958)], leadership style [AOR = 2.19 (1.31–3.65)], and supportive supervision [AOR = 2.05 (1.27–3.32)] were found significant determinants of job satisfaction. The overall level of job satisfaction among health care professionals at the University of Gondar Referral Hospital was low. Therefore, health service managers should focus their leadership style and provide supportive supervision in the hospital to improve the level of job satisfaction of health care professionals.

**Electronic supplementary material:**

The online version of this article (10.1186/s13104-018-3918-0) contains supplementary material, which is available to authorized users.

## Introduction

Health care professionals play a central and critical role in improving access and quality health care for the population. The World Health Organization (WHO) Global strategy on human resources on health workforce 2030 sets out the policy agenda to ensure a workforce that is fit for purpose to attain the targets of the Sustainable Development Goals(SDGs) [1]. Motivation of health care workers can initiate them to exert and maintain an effort towards organizational goals. Motivation depends up on many factors, and job satisfaction is one of the most important factors [2].

The term job satisfaction refers to the attitude and feelings of people about their work. Positive and favorable attitudes towards the job indicate job satisfaction. Whereas, negative and unfavorable attitudes towards their job indicate job dissatisfaction [3]. A high level of job satisfaction has a positive effect on workers’ health related quality of life [4–7], job performance [7–10], retention in work [11–13], quality of healthcare delivery [14, 15] and patient satisfaction [16, 17]. Low job satisfaction may result in staff turnover, tiredness, absenteeism, undesirable job performance and poor quality of service to clients [18–20].

Previous studies have shown that job satisfaction could be positively influenced by several factors such as payment and compensation, good interpersonal relationship, training and career growth, supportive leadership, recognition by management, better teamwork and safe working environment [19, 21–27]. Conversely, job satisfaction could be negatively affected by factors such as work load, work–family conflict, poor doctor–patient relationship, improper supervision, lack of training opportunities, low salaries, and financial rewards [28–30].

In Ethiopia, previous studies [19, 21, 23, 25, 31–34] have reported a varied level of job satisfaction among health care professionals. There is limited evidence regarding this issue in Northwest Ethiopia. Thus, this study aimed to investigate the job satisfaction of health care professionals working in the University of Gondar referral hospital and to explore its associated factors.

## Main text

### Methods

#### Study design, area and period

An institution based cross-sectional study was conducted from March 27, 2017 to April 25, 2017 at the University of Gondar Referral Hospital. The hospital is located 738 km from Addis Ababa, which is the capital city of Ethiopia. It provides a full range of health care services including outpatient, inpatient and surgical services. This hospital is expected to serve for more than 5 million people in its catchment area. The hospital has 1040 health care professionals, 580 beds in five different inpatient departments and 14 wards, and 14 different units giving outpatient services to customers. Besides, this hospital serve as a referral hospital for Northwest Ethiopia [35].

#### Sample size determination and sampling procedure

The required sample size was calculated using a single population proportion formula:

$${\text{n}} = \frac{{\left( {{\text{z}}\upalpha/2} \right)^{2} {\text{p}}\left( {1 - {\text{p}}} \right)}}{{{\text{d}}^{2} }}$$

*Assumptions* n = required sample size, Z = critical value for normal distribution at 95% confidence level (1.96), d = 0.05 (5% margin of error), P = 44.2% (proportion of healthcare professionals satisfied with their job) [25] and an estimated non-response rate of 10%. The final calculated sample size for this study was 416. To select the study participants, first, health care professionals who had a work experience of 6 months and above were included. However, health care professionals who were on maternal or annual leave or those who were seriously ill during data collection period were excluded. Then after, a simple random sampling technique was used to select each professional proportionally from all categories of professions(medical doctor, nurse, midwifery, pharmacy, laboratory, radiologist, physiotherapist, optometrist, environmental health, health officer, dentist, anesthetist, and Psychiatrist) based on the number of professionals in each category.

#### Data collection procedure

Data were collected using a pretested and structured interviewer administered questionnaire (Additional file 1). The questionnaire was prepared in English and translated to Amharic, then back to English to check for its consistency. The reliability of the tool for each subscale was checked using Cronbach’s alpha reliability test, which was 0.83, which showed the consistency of the questionnaire. To assure the data quality, two diploma nurses and one BSc public health professional were recruited as data collectors and supervisor, respectively. In addition, training regarding the study objectives and data collection process was given for data collectors and supervisor for 2 days. Moreover, the questionnaire was pretested among 5% of the sample size at Felege Hiwot referral hospital. Furthermore, intensive supervision was done by supervisor and principal investigators throughout the data collection period.

#### Study variables

The dependent variable of this study was level of job satisfaction. Assessment of Job satisfaction was measured by using twenty items each scored 5-point Likert scale with 1 denoting strongly dissatisfied and 5 denoting strongly satisfied with Minnesota Satisfaction Questionnaire (MSQ) short form [36]. The questions related to factors associated with job satisfaction were prepared by reviewing previous similar studies [27, 37, 38]. The overall job satisfaction was estimated by taking the sum score of all the subscales. Then, to measure the level of job satisfaction of each individual, respondents who scored more than 60 of the sum of all the satisfaction scale items were considered as satisfied with their job. Those who scored 60 and below were taken as dissatisfied [39]. For each domain factors, the sum score of each variable under domains value of 60 was taken as a cut point value to determine whether a health worker satisfied with his/her job or not. As a result, healthcare professionals who scored a value of 60 and below considered as dissatisfied and those who scored greater than 60 were considered as satisfied [39].

The independent variables were: socio-demographic characteristics (age, sex, marital status, education level, profession category, work experiences, salary and alternative job), intrinsic motivator factors(achievement, advancement, recognition and reward, growth and work itself or nature of work) and extrinsic or hygienic factors (benefit and payment, supervision support, organizational policy and strategy, work environment, staff relationship, and work security).

#### Data processing and analysis

Data were cleaned, coded and entered using Epi-Info software Version 7 and analyzed using SPSS Version 20. Mean, mode, and median were used for continuous variables whereas; percentage was used for categorical variables. Descriptive results were presented using tables and figures. Model fitness was checked using a Hosmer–Lemeshow goodness-of-fitness test. Crude odds ratios with their 95% confidence intervals were estimated in the bivariable logistic regression analysis to assess the association between each independent variable and outcome variable. In the bivariable logistic regression, variables with P-value < 0.2 were fitted into the multivariable logistic regression analysis. Finally, adjusted odds ratios with their 95% confidence intervals were estimated to assess the strength of association, and variables with P-value < 0.05 were considered statistically significant factors.

### Results

#### Socio-demographic characteristics of the study participants

A total of 383 health care professionals were included in the study, resulting in a response rate of 92.1%. About two hundred twenty–three (58.2%) and more than half (53.5%) of the respondents were males and unmarried, respectively. The median age of participants was 28 (IQR 25–32) years. Majority (79.9%) and nearly half (49.6%) of the respondents had a bachelor degree and 1–5 years work experience, respectively. The median monthly salary of the respondents was 6179 (IQR4446-7111) Ethiopian Birr. Three-fourth (75.2%) of them had no alternative job opportunities (Table 1).

**Table 1:** Socio-demographic characteristics of Health care professionals working at University of Gondar Referral Hospital, Northwest Ethiopia, 2017 (n = 383)

Variables	Category	Frequency	Percentage
Sex	Male	223	58.2
	Female	160	41.8
Age	Less than 30	153	39.9
	30 and above	230	60.1
Marital status	Unmarried	205	53.5
	Married	178	46.5
Educational status	Diploma	50	13.1
	Degree	306	79.9
	Above degree	27	7.0
Profession category	Medical doctor	52	13.6
	Nurse	164	42.8
	Midwifery	37	9.7
	Pharmacy	31	8.1
	Laboratory	46	12
	Optometrist	34	8.9
	Others^a^	19	4.9
Work experiences	Less than 1 year	50	13.1
	1–5 years	190	49.6
	6–10 years	107	27.9
	Above 10 years	36	9.4
Salary (ETB)	Less than 4446	119	31.1
	4446–6179	133	34.7
	6180–7111	43	11.2
	Above 7111	88	23
Alternative job opportunities	Yes	94	25
	No	288	75

^a^Radiologist, Physiotherapist, Environmental health, Health officer, Dentist, Anesthetist, and Psychiatrist

#### Level of job satisfaction

**Fig. 1:**
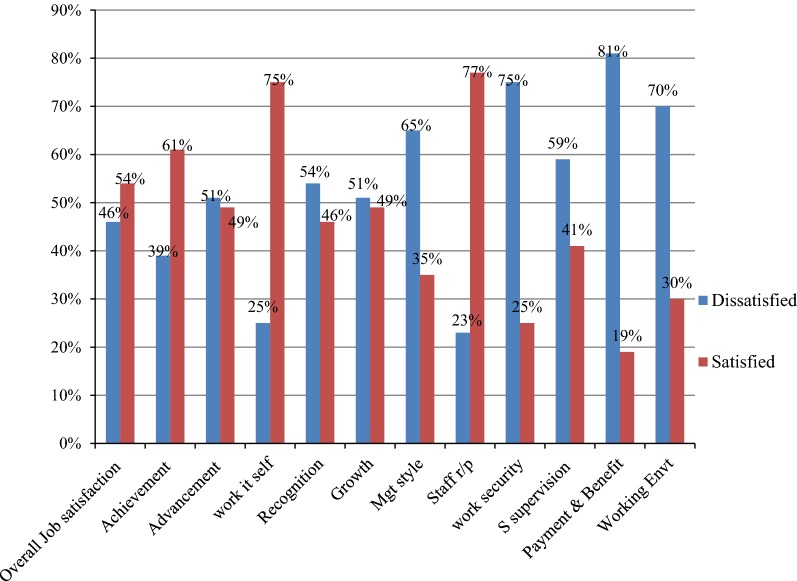
Level of job satisfaction among healthcare professionals at University of Gondar Referral Hospital, Northwest Ethiopia, 2017

In this study, the overall prevalence of job satisfaction among health care professionals at the University of Gondar Referral Hospital was 54% (95% CI 49.3, 58.8%). The highest level of job satisfaction score among domain factors was observed on staff relationship (77%) and the work itself (75%) (Fig. 1).

#### Factors associated with job satisfaction

A multivariable binary logistic regression analysis was performed to identify factors associated with job satisfaction of health care professionals. Consequently, four variables were found to be statistically associated with job satisfaction after adjusting for confounders. These were marital status, salary, leadership style, and supportive supervision. In this study, married health care professionals were 1.79 times more likely to be satisfied by their job as compared to unmarried health care professionals [AOR = 1.79; 95% CI (1.14, 2.79)]. Health care professionals who had a monthly salary above 6179 ETB were 2.75 times more likely to be satisfied with their job as compared to those who had salary income 6179 ETB and less [AOR = 2.75; 95% CI (1.27–5.96)]. Study participants who experienced democratic way of leadership style from their manager were 2.19 times more likely to be satisfied with their job as compared to those who experienced autocratic style of leadership from their manager [AOR 2.19; 95% CI (1.31–3.65)]. Those respondents who got adequate supportive supervision were 2.05 times more likely to be satisfied with their job as compared to those who did not get adequate supportive supervision for their work [AOR 2.05; 95% CI (1.28–3.32)] (Table 2).

**Table 2:** Bivariable and multivariable logistic regression analysis of factors with job satisfaction among health care professionals working at University of Gondar Referral hospitals, Ethiopia 2017 (n = 383)

Factors	Category	Job satisfaction			
		Satisfied	Unsatisfied	COR (95% CI)	AOR (95% CI)
Age (years)	< 30	94	59	1.62 (1.07, 2.46)	0.735 (0.42, 1.28)
	30 and above	114	116	1	1
Marital status	Married	107	71	1.55 (1.03, 2.33)*	1.79 (1.14, 2.79)*
	Unmarried	101	104	1	1
Salary in ETB	Above 6179	83	48	1.76 (1.14, 2.71)*	2.75 (1.27, 5.96)*
	≤ 6179	125	127	1	1
Work achievement	Good	144	90	2.13 (1.40, 3.23)**	1.17 (0.70, 1.95)
	Poor	64	85	1	1
Advancement	Yes	120	67	2.19 (1.46, 3.31)*	1.29 (0.76, 2.17)
	No	88	108	1	1
Work itself	Pleasant	168	120	1.93 (1.20, 3.08)**	0.89 (0.51, 1.60)
	Unpleasant	40	55	1	
Recognition	Yes	112	64	2.02 (1.34, 3.05)*	1.33 (0.81, 2.19)
	No	96	111	1	1
Growth	Yes	119	68	2.10 (1.40, 3.17)**	1.03 (0.62, 1.71)
	No	89	107	1	1
Leadership style	Democratic	93	40	2.73 (1.75, 4.27)**	2.19 (1.31, 3.65)*
	Autocratic	115	135	1	1
Work security	Yes	66	28	2.44 (1.48, 4.02)*	1.309 (0.72, 2.39)
	No	142	147	1	1
Supportive supervision	Adequate	107	50	2.65 (1.73, 4.05)**	2.05 (1.27, 3.32)*
	Inadequate	101	125	1	1
Payment and benefit	Fair	52	21	2.44 (1.41, 4.25)*	1.48 (0.78, 2.83)
	Unfair	156	154	1	1
Safe working environment	Yes	76	40	1.94 (1.24, 3.05)*	1.03 (0.60, 1.76)
	No	132	135	1	1

*ETB* Ethiopian Birr, *CI* confidence interval, *COR* crude odds ratio, *AOR* adjusted odds ratio

* P < 0.05, ** P < 0.001

### Discussion

In this study, the overall level of job satisfaction among health care professionals was 54% (95% CI 49.3, 58.8%). This finding is comparable with previous studies conducted in Ethiopia (52.9% in Addis Ababa [21], and 54.2% in East Gojjam Zone [31]) and India (50%) [40]. But, our finding is lower than satisfaction rate reported in Nigeria (90.4%) [41], Nepal (76%) [22], Eastern India (59.6%) [42] and Spain (77.2%) [43]. The possible explanation for the above difference could be due to the difference in socio-economic characteristics and organizational set-up of health care workers. On the other hand, our finding is higher than studies done in Pakistan (41%) [28], Sri Lanka (23.7%) [44], Turkey (45.5%) [24] and in Ethiopia: West Showa Zone (34.9%) [32], Addis Ababa (43.2%) [19], Western Amhara (31.7%) [34], Harar (44.2%) [25], Northwest Ethiopia (46.9%) [45] and West Ethiopia (41.46%) [23]. The possible reasons for this variation might be due to the difference in study population, setting, and time. Another possible explanation for the above variation could be due to the difference in the tools used to measure the outcome variable.

Regards to determinants of job satisfaction, this study has found out that the factors significantly associated were salary, marital status, leadership style and supportive supervision. Health professionals paid high salary were found to be more satisfied with their job as compared to their counterparts. This finding is supported by other studies conducted elsewhere [23, 24, 45–48]. In addition, this study has found that married health professionals were more likely to be satisfied with their job as compared to their unmarried counterparts. This finding is consistent with studies reported from elsewhere [21, 40, 49]. However, a study conducted in Nepal has found that no such difference was observed between married and unmarried health care professionals [22]. It is possible that married couples are more likely to help each other socially, psychologically and in economic terms.

In relation to supportive supervision, health care professionals who got adequate support supervision in their work were more likely to be satisfied as compared to those who did not get adequate support. This finding is in line with a three country study in Africa [50]. This could be explained by the fact that adequate and effective supportive supervision can motivate staffs leading satisfaction on their job. A negative/critical rather than supportive or an absence of workplace supervision leads dissatisfaction of workers at their job.

Finally, health care professionals who reported a democratic leadership style were more likely to be satisfied with their job than their counterparts. This is consistent with a study in USA [51]. The possible explanation might be due to the fact that workers with democratic way of leadership style might get an opportunity to participate in any decision making process concerning their job. This study revealed a low level of satisfaction of health care professionals. Job satisfaction was significantly associated with marital status, salary, leadership style, and supportive supervision. Therefore, the study hospital manager should give a special emphasis on leadership style, supervision, and salary of health care workers to increase their job satisfaction. Future longitudinal studies should be conducted to identify factors that enhance job satisfaction for the hospital health professionals.

## Limitations of the study

Due to the cross-sectional nature of this study, establishing a true cause and effect relationship between job satisfaction and associated factors would be impossible. Since our participants were limited to health workers in University of Gondar referral hospital, the generalization of our findings could be difficult. In addition, we didn’t separately study the job satisfaction across each category of healthcare professionals which might affect a clearer picture of the relationship between job satisfaction and profession.

## Additional file

**Additional file 1.** English version questionnaire.

## Abbreviations

WHO: World Health Organization
SDGs: Sustainable Development Goals
ETB: Ethiopian Birr
USA: United States of America
CI: Confidence Interval
COR: Crude Odds Ratio
AOR: Adjusted Odds Ratio

## References

[1] World Health Organization. Health workforce requirements for universal health coverage and the Sustainable Development Goals. Hum Resour Health Obs. 2016;17.

[2] Luoma M. Increasing the motivation of health care workers. Capac Proj Tech Brief. 2006;7.

[3] Aziri B. Job satisfaction: a literature review. Manag Res Pract 2011;3(4).

[4] Cass MH, Siu OL, Faragher EB, Cooper CL (2003). A meta-analysis of the relationship between job satisfaction and employee health in Hong Kong. Stress Health J Int Soc Invest Stress.

[5] Faragher E. Brian, Cass M., Cooper Cary L. (2013). The Relationship between Job Satisfaction and Health: A Meta-Analysis. From Stress to Wellbeing Volume 1.

[6] Ioannou P, Katsikavali V, Galanis P, Velonakis E, Papadatou D, Sourtzi P (2015). Impact of job satisfaction on Greek nurses’ health-related quality of life. Saf Health Work.

[7] Rahman M, Sen A (1987). Effect of job satisfaction on stress, performance and health in self-paced repetitive work. Int Arch Occup Environ Health.

[8] Bakotić D (2016). Relationship between job satisfaction and organisational performance. Econ Res Ekonomska istraživanja.

[9] Platis C, Reklitis P, Zimeras S (2015). Relation between job satisfaction and job performance in healthcare services. Procedia-Soc Behav Sci.

[10] Shakeri MT (2014). The relationship between job satisfaction and job performance among midwives working in healthcare centers of Mashhad, Iran. Reprod Health.

[11] Biason RS. The effect of job satisfaction to employee retention.

[12] Lichtenstein RL (1984). The job satisfaction and retention of physicians in organized settings: a literature review. Med Care Rev.

[13] Santhanam G, Jayaraman R, Badrinath V: Influence of perceived job satisfaction and its impacts on employee retention in Gulf Cooperation Countries. In: Proceedings of 2012 international conference on management issues in emerging economies (ICMIEE), conference 2012. Piscataway: IEEE; 2012. p. 69–73.

[14] Aron S. Relationship between nurses’ job satisfaction and quality of healthcare they deliver. 2015.

[15] Farman A, Kousar R, Hussain M, Waqas A, Gillani SA. Impact of job satisfaction on quality of care among nurses on the public hospital of Lahore, Pakistan.

[16] Janicijevic I, Seke K, Djokovic A, Filipovic T (2013). Healthcare workers satisfaction and patient satisfaction—where is the linkage?. Hippokratia.

[17] Szecsenyi J, Goetz K, Campbell S, Broge B, Reuschenbach B, Wensing M (2011). Is the job satisfaction of primary care team members associated with patient satisfaction?. BMJ Qual Saf.

[18] Marinucci F, Majigo M, Wattleworth M, Paterniti AD, Hossain MB, Redfield R (2013). Factors affecting job satisfaction and retention of medical laboratory professionals in seven countries of Sub-Saharan Africa. Hum Resour Health.

[19] Tadese T, Mohamed A, Mengistie A. Assessment of factors influencing job satisfaction among health care providers, federal police referral hospital, Addis Ababa, Ethiopia. Ethiop J Health Dev (EJHD). 2016;29(2).

[20] Yami A, Hamza L, Hassen A, Jira C, Sudhakar M. Job satisfaction and its determinants among health workers in Jimma university specialized hospital, southwest Ethiopia. Ethiop J Health Sci. 2011;21(3). PMC3275875 22435005

[21] Bekru ET, Cherie A, Anjulo AA (2017). Job satisfaction and determinant factors among midwives working at health facilities in Addis Ababa city, Ethiopia. PLoS ONE.

[22] Chaulagain N, Khadka DK (2012). Factors influencing job satisfaction among healthcare professionals at Tilganga eye centre, Kathmandu, Nepal. Int J Sci Technol Res.

[23] Deriba BK, Sinke SO, Ereso BM, Badacho AS (2017). Health professionals’ job satisfaction and associated factors at public health centers in West Ethiopia. Hum Resour Health.

[24] Eker L, Tüzün EH, Daskapan A, Sürenkök Ö (2004). Predictors of job satisfaction among physiotherapists in Turkey. J Occup Health.

[25] Geleto A, Baraki N, Atomsa GE, Dessie Y (2015). Job satisfaction and associated factors among health care providers at public health institutions in Harari region, eastern Ethiopia: a cross-sectional study. BMC Res Notes.

[26] Khamlub S, Harun-Or-Rashid M, Sarker MAB, Hirosawa T, Outavong P, Sakamoto J (2013). Job satisfaction of health-care workers at health centers in Vientiane Capital and Bolikhamsai Province, Lao PDR. Nagoya J Med Sci.

[27] Schwendimann R, Dhaini S, Ausserhofer D, Engberg S, Zúñiga F (2016). Factors associated with high job satisfaction among care workers in Swiss nursing homes—a cross sectional survey study. BMC Nurs.

[28] Kumar R, Ahmed J, Shaikh BT, Hafeez R, Hafeez A (2013). Job satisfaction among public health professionals working in public sector: a cross sectional study from Pakistan. Hum Resour Health.

[29] Lu Y, Hu X-M, Huang X-L, Zhuang X-D, Guo P, Feng L-F, Hu W, Chen L, Hao Y-T (2016). Job satisfaction and associated factors among healthcare staff: a cross-sectional study in Guangdong Province, China. BMJ Open.

[30] Shi L, Song K, Rane S, Sun X, Li H, Meng Q (2014). Factors associated with job satisfaction by Chinese primary care providers. Prim Health Care Res Dev.

[31] Gualu Dessalegn Haile Tenaw, Zeleke Haymanot, Dessalegn Berhanu (2017). Job satisfaction and associated factors among nurses in East Gojjam Zone Public Hospitals Northwest Ethiopia. J Nurs Care..

[32] Mekuria M, Geleto A (2015). Factors associated to job satisfaction among healthcare workers at public hospitals of West Shoa Zone, Oromia Regional State, Ethiopia: a cross sectional study. J Sci Publ Group.

[33] Mengistu MM, Bali AG (2015). Factors associated job satisfaction among health care workers at public hospitals of west Shoa Zone, Oromia, Regional staff, Ethiopia: a cross-sectional study. Sci J Public Health.

[34] Temesgen K, Aycheh MW, Leshargie CT (2018). Job satisfaction and associated factors among health professionals working at Western Amhara Region, Ethiopia. Health Qual Life Outcomes.

[35] Hospital UoGR: Planning, monitoring and evaluation department report. 2017.

[36] Minnesota Satisfaction Questionnaire (MSQ) Short form. https://sites.uni.edu/butlera/courses/org/msq.htm. Accessed 17 Aug 2018.

[37] Baylor KM. The influence of intrinsic and extrinsic job satisfaction factors and affective commitment on the intention to quit for occupations characterized by high voluntary attrition. 2010.

[38] Kuzey C (2016). Impact of health care employees’ job satisfaction on organizational performance support vector machine approach. Eur J Econ Polit Stud.

[39] Dachew BA, Birhanu AM, Bifftu BB, Tiruneh BT, Anlay DZ (2016). High proportion of intention to leave among academic staffs of the University of Gondar, Northwest Ethiopia: a cross-sectional institution-based study. Int J Innov Med Educ Res.

[40] RAshid SSM. Determinants of job satisfaction among nurses at the Muhimbili National Hospital 2013.

[41] Kolo ES (2018). Job satisfaction among healthcare workers in a tertiary center in kano, Northwestern Nigeria. Niger J Basic Clin Sci.

[42] Bhattacherjee S, Ray K, Roy JK, Mukherjee A, Roy H, Datta S (2016). Job satisfaction among doctors of a government medical college and hospital of Eastern India. Nepal J Epidemiol.

[43] Carrillo-García C, Solano-Ruíz MDC, Martínez-Roche ME, Gómez-García CI (2013). Job satisfaction among health care workers: the role of gender and age. Revista latino-americana de enfermagem.

[44] Dilina SCGaH: Employee satisfaction and related factors among public healthcare workers in Sri Lanka: a case study on Regional Directorate of Hambanthota. JOJ Nurs Health Care. 2018;8(4).

[45] Yilkal Fentie D, Enyew Ashagrie H, Getinet Kasahun H. Job satisfaction and associated factors among anesthetists working in Amhara National Regional State, Northwest Ethiopia, May 2017: a multicenter cross-sectional study. Anesthesiol Res Pract. 2018;2018. 10.1155/2018/6489674 PMC5954967 29853872

[46] Ayamolowo SJ (2013). Job satisfaction and work environment of primary health care nurses in Ekiti State, Nigeria: an exploratory study. Int J Caring sci.

[47] Jaiswal P, Gadpayle A, Singhal AK, Sachdeva S, Modi RK, Padaria R, Ravi V (2015). Job satisfaction among hospital staff working in a Government teaching hospital of India. Med J Dr DY Patil Univ.

[48] Tasneem Saima, Cagatan Ayse Seyer., Avci Mehmet Zeki., Basustaoglu Ahmet Celal. (2018). Job Satisfaction of Health Service Providers Working in a Public Tertiary Care Hospital of Pakistan. The Open Public Health Journal.

[49] Jathanna R, Melisha R, Mary G, Latha K. Determinants of job satisfaction among healthcare workers at a tertiary care hospital. Online J Health Allied Sci. 2011;10(3).

[50] McAuliffe E, Daly M, Kamwendo F, Masanja H, Sidat M, de Pinho H (2013). The critical role of supervision in retaining staff in obstetric services: a three country study. PLoS ONE.

[51] Jones WL. Leadership styles and nursing satisfaction rates. 2011.

