# Correction to: Level of job satisfaction and associated factors among health care professionals working at University of Gondar Referral Hospital, Northwest Ethiopia: a cross-sectional study

**DOI:** 10.1186/s13104-018-3972-7

**Published:** 2018-12-04

**Authors:** Getnet Gedif, Yetnayet Sisay, Animut Alebel, Yihalem Abebe Belay

**Affiliations:** 1 Department of Public Health, College of Health Sciences, Debre Markos University, P.O.BOX:269, Debre Markos, Ethiopia; 2 Department of Nursing, College of Health Sciences, Debre Markos University, Debre Markos, Ethiopia; 3 Department of Health Education and Behavioral Science, Institute of Public Health, University of Gondar, Gondar, Ethiopia

## Correction to: BMC Res Notes (2018) 11:824 10.1186/s13104-018-3918-0

Following publication of the original article [1], the authors reported that one of the authors’ names was spelled incorrectly. In this Correction the incorrect and correct author name are shown. The original publication of this article has been corrected.

Originally the author name was published as:

- Genet Gedif

The correct author name is:

- Getnet Gedif
