# Supplementary material for: Level of job satisfaction and associated factors among health care professionals working at University of Gondar Referral Hospital, Northwest Ethiopia: a cross-sectional study
Source: BMC Res Notes. 2018 Nov 20;11:824. doi: 10.1186/s13104-018-3918-0 (PMC6245915; doi:10.1186/s13104-018-3918-0)
Supplement: Supplementary file 1 — Additional file 1. English version questionnaire. [file 13104_2018_3918_MOESM1_ESM.docx]

## Annex-1 English Version Questionnaire

For Health Professionals Working at Gondar University Referral Hospital, Northwest, Ethiopia

Working unit _____________________ code ________ Participant code ________

Data collector name _______________date ____________signature __________

**Part I- Socio-demographic characteristics of the respondents**

| \| No \|  \|  \|  \| \| --- \| --- \| --- \| --- \| | Questions | Responses | Remark |
| --- | --- | --- | --- | --- | --- | --- | --- |
| 101 | Your sex | 1. Male 2. Female |  |
| 102 | Age in year | ---------- |  |
| 103 | Marital status | 1.Single 2.Married  3.Divorced 4.Widowed |  |
| 104 | Educational Status | 1. Diploma 2. BSc  3. MSc or MPH  4. Others(specify)---------------- |  |
| 105 | Profession | 1. Medical Dr. 5. Pharmacy  2. Nurse 6. Laboratory  3. Midwifery 7. Optometrist  4. others(Specify)------------------ |  |
| 106 | Total Work experiences | ------- year & -------- month |  |
| 107 | Salary in birr | --------------------- |  |
| 108 | Do you have alternative job opportunities? | 1. Yes 2. No |  |

**Part II: Job satisfaction factors questionnaire**

**Instruction:** This section of the questionnaire asks for your opinion about whether you agreed or not at this facility. Each statement has five alternatives with five point scale. Please circle the one number for each question that comes closest to reflecting your opinion about it. (Strongly agree (SA) =5, agree (A) =4, neither agree nor disagree (N) =3,disagree (D) =2, strongly disagree (SD) =1)

| S. N | **Motivation /Intrinsic factor /** | | | Possible answers | | | | | |
| --- | --- | --- | --- | --- | --- | --- | --- | --- | --- |
| 1 | Achievement | | |  | | | | | |
| 109 | I am proud to work in this health institution because it recognizes my achievements | | | 1 | 2 | 3 | 4 | | 5 |
| 110 | I feel satisfied with my job because it gives me feeling of accomplishment. | | | 1 | 2 | 3 | 4 | | 5 |
| 111 | I feel i have contributed towards my health institution in a positive manner. | | | 1 | 2 | 3 | 4 | | 5 |
| 2 | Advancement | | |  | | | | | |
| 112 | I will choose career advancement rather than monetary incentives. | | | 1 | 2 | 3 | 4 | | 5 |
| 113 | My job allows me to learn new skills for career advancement. | | | 1 | 2 | 3 | 4 | | 5 |
| 114 | The fairness wether it is based on job performance/ achievement) | | | 1 | 2 | 3 | 4 | | 5 |
| 3 | **Work itself /Nature of the work** | | |  | | | | | |
| 115 | My work is thrilling and i have a lot of variety in tasks that i do. | | | 1 | 2 | 3 | 4 | | 5 |
| 116 | I am empowered enough to do my job. | | | 1 | 2 | 3 | 4 | | 5 |
| 117 | I feel happy by the chance of using my full capacity to do my job | | | 1 | 2 | 3 | 4 | | 5 |
| 4 | **Recognition** | | |  |  |  |  | |  |
| 118 | My manager always thanks me for a job well done. | | | 1 | 2 | 3 | 4 | | 5 |
| 119 | I receive adequate recognition for doing my job well | | | 1 | 2 | 3 | 4 | | 5 |
| 5 | **Growth** | | |  |  |  |  | |  |
| 120 | I am proud to work in my health institution because i feel i have grown as a person | | | 1 | 2 | 3 | 4 | | 5 |
| 121 | My job allows me to improve my experience, skills and performance | | | 1 | 2 | 3 | 4 | | 5 |
|  | **Hygienic / Extrinsic factors** | | | | | | | | |
| 6 | **Organizational policy** | | | | | | | | |
| 122 | The attitude of the administration is accommodative in my Health institution | 1 | 2 | | 3 | 4 | | 5 | |
| 123 | I am proud being here due to the health institution policy is favorable for workers | 1 | 2 | | 3 | 4 | | 5 | |
| 124 | I completely understand the mission of my health institution | 1 | 2 | | 3 | 4 | | 5 | |
| 7 | **Relationship** |  |  | |  |  | |  | |
| 125 | It is easy to get along with my colleagues | 1 | 2 | | 3 | 4 | | 5 | |
| 126 | My colleagues are helpful and friendly | 1 | 2 | | 3 | 4 | | 5 | |
| 8 | **Work security** |  |  | |  |  | |  | |
| 127 | I believe safe working at my workplace | 1 | 2 | | 3 | 4 | | 5 | |
| 128 | My workplace is located in an area where i feel comfortable | 1 | 2 | | 3 | 4 | | 5 | |
| 129 | Medical supplies and equipment’s are adequately available | 1 | 2 | | 3 | 4 | | 5 | |
| 9 | **Relationship With supervisor** |  |  | |  |  | |  | |
| 130 | I feel my performance has improved because of the support from my supervisor | 1 | 2 | | 3 | 4 | | 5 | |
| 131 | I feel satisfied at work because of my relationship with my supervisor | 1 | 2 | | 3 | 4 | | 5 | |
| 132 | My supervisors are strong and trustworthy leaders | 1 | 2 | | 3 | 4 | | 5 | |
| 10 | **Payment and Benefit** |  |  | |  |  | |  | |
| 133 | I believe my salary is fair | 1 | 2 | | 3 | 4 | | 5 | |
| 134 | my payment is favorable when compared to other organization's | 1 | 2 | | 3 | 4 | | 5 | |
| 135 | I am encouraged to work harder because of my Salary | 1 | 2 | | 3 | 4 | | 5 | |
| 11 | **Working Condition/Environment /** |  |  | |  |  | |  | |
| 136 | I feel satisfied because of the support(comfort) i am provided at work | 1 | 2 | | 3 | 4 | | 5 | |
| 137 | I am proud to work because of the pleasant working conditions | 1 | 2 | | 3 | 4 | | 5 | |

**PARTIII: Minnesota Satisfaction Questionnaire (MSQ) ፡**

This section of the questionnaire asks for your opinion about whether you satisfied or not at this facility with the intrinsic and extrinsic factors.

Ask yourself: How **satisfied** am I with this aspect of my job?

1= If you are **very dissatisfied** about the statement.

2= If you are **dissatisfied** about the statement.

3= If you are **neither satisfied** nor **dissatisfied (neutral)** about the statement.

4= If you are **satisfied** about the statement.

5= If you **very satisfied** about the statement

Please circle the one number for each question that comes closest to reflecting your opinion about it.

| No. | Job satisfaction factor | 1=Very  dissatisfied | 2=Dissatisfied | 3=Neutral | 4=Satisfied | 5=Very  Satisfied |
| --- | --- | --- | --- | --- | --- | --- |

| 138 | Being able to keep busy all the time. | 1 | 2 | 3 | 4 | 5 |
| --- | --- | --- | --- | --- | --- | --- |
| 139 | The chance to work alone on the job. | 1 | 2 | 3 | 4 | 5 |
| 140 | The chance to do alternative from to time. | 1 | 2 | 3 | 4 | 5 |
| 141 | The chance to be ―somebody ―in the community. | 1 | 2 | 3 | 4 | 5 |
| 142 | The way my boss handle his / her workers. | 1 | 2 | 3 | 4 | 5 |
| 143 | The competence of my supervisor in making decision. | 1 | 2 | 3 | 4 | 5 |
| 144 | Being able to do things that don‘t go against my conscious | 1 | 2 | 3 | 4 | 5 |
| 145 | The way my job provides for steady employment. | 1 | 2 | 3 | 4 | 5 |
| 146 | The chance to be responsible for the work of others | 1 | 2 | 3 | 4 | 5 |
| 147 | The chance to tell people what to do. | 1 | 2 | 3 | 4 | 5 |
| 148 | The chance to do something that makes use of my abilities. | 1 | 2 | 3 | 4 | 5 |
| 149 | The way company policies are put into practice. | 1 | 2 | 3 | 4 | 5 |
| 150 | My pay and the amount of work I do. | 1 | 2 | 3 | 4 | 5 |
| 151 | The chances for advancement on this job. | 1 | 2 | 3 | 4 | 5 |
| 152 | The freedom to use my own judgment. | 1 | 2 | 3 | 4 | 5 |
| 153 | The chance to try my own methods of doing the job | 1 | 2 | 3 | 4 | 5 |
| 154 | The working condition. | 1 | 2 | 3 | 4 | 5 |
| 155 | The way my co-workers get each other | 1 | 2 | 3 | 4 | 5 |
| 156 | The praise I get for doing a good job. | 1 | 2 | 3 | 4 | 5 |
| 157 | The feeling of accomplishment I get from the job. | 1 | 2 | 3 | 4 | 5 |

**We appreciate your cooperativeness!!!**
